# Adjuvant radiation therapy alone is associated with improved overall survival compared to hormonal therapy alone in older women with estrogen receptor positive early stage breast cancer

**DOI:** 10.1002/cam4.3443

**Published:** 2020-09-17

**Authors:** Sachin R. Jhawar, Naomi Alpert, Emanuela Taioli, Mutlay Sayan, Jose Bazan, Ko Un Park, Daniel Stover, Mathew Cherian, Julia White, Bruce Haffty, Nisha Ohri

**Affiliations:** ^1^ Department of Radiation Oncology Ohio State University Comprehensive Cancer Center Columbus OH USA; ^2^ Institute for Translational Epidemiology and Department of Population Health Science and Policy Icahn School of Medicine at Mount Sinai New York NY USA; ^3^ Department of Radiation Oncology Rutgers Cancer Institute of New Jersey New Brunswick NJ USA; ^4^ Department of Surgical Oncology Ohio State University Comprehensive Cancer Center Columbus OH USA; ^5^ Department of Internal Medicine Ohio State University Comprehensive Cancer Center Columbus OH USA

**Keywords:** breast cancer, breast conserving surgery, early stage, hormone therapy, node negative, radiation

## Abstract

**Background:**

Breast conserving surgery (BCS) and adjuvant hormonal therapy (HT) without radiation therapy (RT) is an acceptable approach for older women with early stage, estrogen receptor (ER) positive breast cancer. Toxicity and compliance remain issues with HT. Adjuvant RT alone may have better compliance, but its efficacy in the absence of HT is unclear. We aim to assess patterns of adjuvant therapy and survival outcomes among older women with early stage, ER positive (ER+) breast cancer.

**Methods:**

The National Cancer Data Base (NCDB) was used to identify 130,194 women age ≥65 years with invasive ER+, node negative breast cancer diagnosed between 2004 and 2015. All patients underwent BCS. Kaplan‐Meier survival curves were used to examine overall survival (OS). The association between adjuvant therapy and OS was assessed in multivariable Cox proportional hazards regression models.

**Results:**

Unadjusted 5/10‐year OS rates were 90.0%/64.3% for HT and RT, 84.2%/54.9% for RT alone, 78.7%/44.5% for HT alone, and 71.6%/38.0% for no treatment; p<0.001 for all. Compared to HT alone, the 10‐year multivariable hazard ratio (HR) for death for RT alone was 0.86 (95% CI 0.82‐0.91). In propensity‐matched patients who received RT alone or HT alone (n=21,326), RT alone had significantly better survival at 5 (HR_adj_: 0.84) and 10 (HR_adj_: 0.87) years.

**Conclusions:**

Older women with early stage ER+ breast cancer who undergo BCS and receive both HT and RT have the best survival, while RT as single‐modality therapy had higher rates of OS at 5 and 10 years compared to HT alone.

## Graphical abstract

In this cohort study of 130,194 women age ≥65 years with invasive ER+, node negative breast cancer who underwent breast conserving surgery, radiation (RT) with hormonal therapy (HT) was associated with the highest 5 and 10‐year survival rates (90.0% and 64.3%, respectively). Compared to patients receiving HT alone, those receiving RT alone had improved unadjusted and adjusted survival rates.

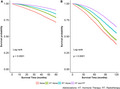

## 1 INTRODUCTION

Hormone receptor positive early stage breast cancer is a highly curable disease, with five‐year disease‐specific survival rates approaching 100% with standard of care therapies in the most favorable subsets.^1^ This has led to questions regarding de‐escalation of therapy, particularly among older patients. Multiple randomized clinical trials have assessed the role of foregoing radiation therapy (RT) in older women with early stage disease who will undergo hormone therapy (HT). These data consistently demonstrate a significant local control benefit with RT but, likely due to competing risks in elderly populations, do not demonstrate a survival benefit.^2^, ^3^, ^4^, ^5^, ^6^ Adjuvant HT alone has therefore been incorporated into the National Comprehensive Cancer Networks (NCCN) guidelines as a category 1 recommendation for women 70 years or older with early stage estrogen‐receptor positive breast cancer.^7^ However, an important consideration is compliance with long‐term HT. Particularly in older women with co‐morbid conditions, there is significant evidence of decreased compliance with completion of five years of adjuvant HT.^8^, ^9^, ^10^

Interest in the omission of RT stems, in part, from the inconvenience of daily treatments. Conventionally fractionated radiation courses were typically delivered daily over 5‐6 weeks. More recently, however, the American Society for Radiation Oncology (ASTRO) updated their practice guidelines and endorsed the use of a hypofractionated course of radiotherapy for the majority of women with early stage breast cancer receiving whole breast irradiation.^11^ Hypofractionated whole breast radiotherapy is delivered over 3‐4 weeks, which can make treatment much more convenient and accessible for patients.

In clinical practice, it is not uncommon that patients desire the benefits of adjuvant therapy to reduce local recurrence risk but are wary of the side effects of hormonal therapy and its prolonged treatment course. This leads to the question of whether radiation therapy alone has a role in the adjuvant management of older patients with early stage breast cancer. In the absence of randomized data to answer this important clinical question, this study uses the National Cancer Data Base (NCDB) to assess patterns of care in older patients with early stage breast cancer and compare survival outcomes based on adjuvant therapy.

## 2 METHODS

### 2.1 Data source

The National Cancer Data Base (NCDB) is a joint project between the American College of Surgeons and the American Cancer Society, which captures approximately 70% of newly diagnosed cancers in the United States each year.^12^ Patients who receive any of their cancer care at a Commission on Cancer (CoC)‐accredited facility are included in the NCDB. The NCDB includes data on cancer characteristics, patient demographics, facility characteristics, first course of treatment, and survival.^13^, ^14^ Because the data used in this study were extracted from the de‐identified NCDB file, research was considered exempt from institutional review board approval. The American College of Surgeons and the Commission on Cancer have not verified and are not responsible for the analytic or statistical methodology employed, or the conclusions drawn from these data by the investigator.

### 2.2 Study population

**Figure 1:**
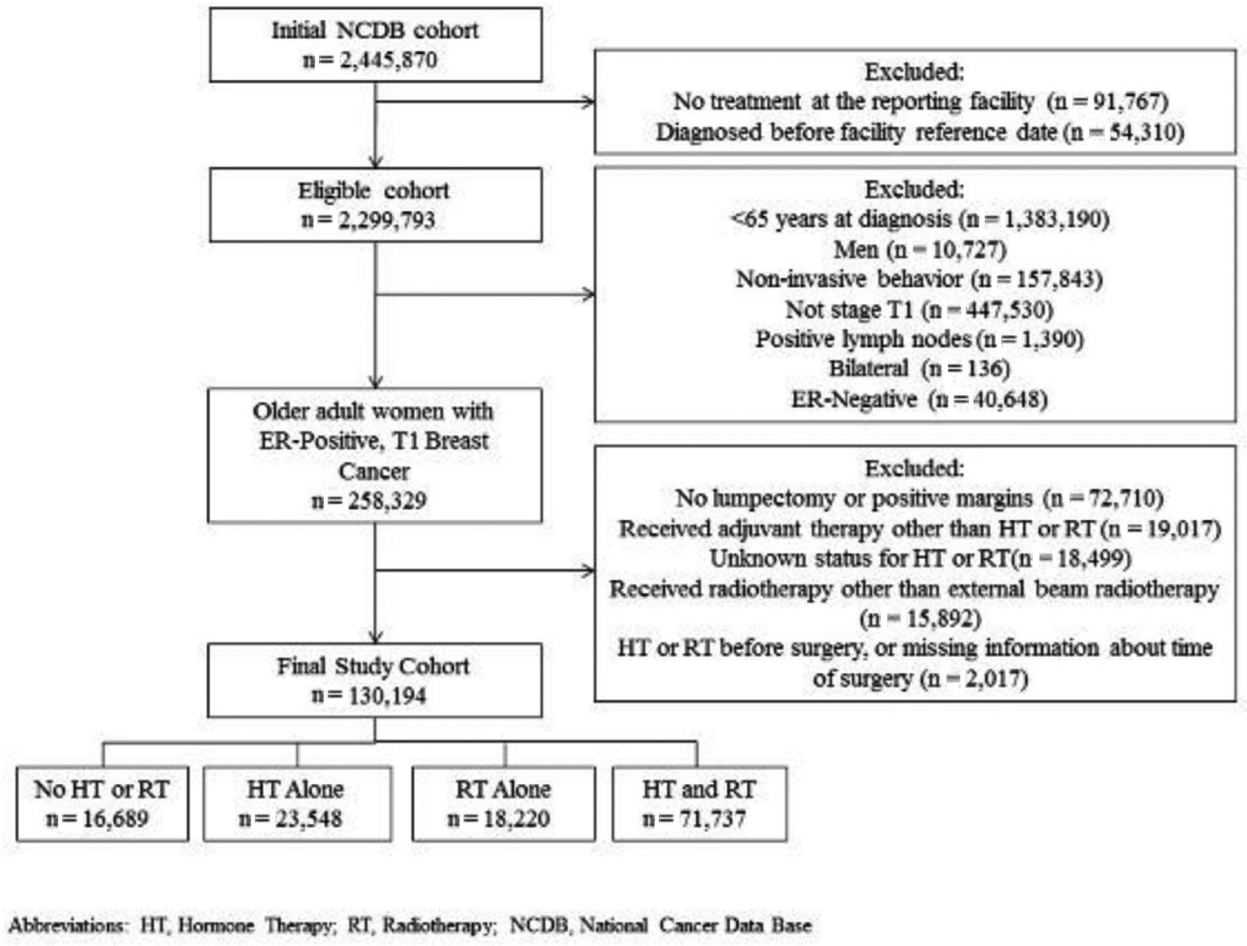
Selection criteria

The initial NCDB dataset included 2,445,870 patients diagnosed with breast cancer from 2004‐2015. As recommended by the NCDB, only patients who received treatment at the reporting facility and whose diagnosis followed the reporting facility’s reference data for data completeness were included (n=2,299,793). These data were queried for women at least 65 years old who received a diagnosis of invasive, node negative, T1, ER‐positive, unilateral breast cancer (n=258,329) and underwent a lumpectomy with negative margins (n=185,619). Those who 1) received adjuvant chemotherapy or immunotherapy; 2) had unknown status for hormone therapy (HT) or radiotherapy (RT) or received RT other than external beam radiotherapy (EBRT); or 3) received HT or RT prior to surgery were excluded (n=55,425), yielding a sample of 130,194 women (Figure 1).

### 2.3 Outcomes and predictor variables

The primary predictor was the type of adjuvant treatment received. Adjuvant HT was defined if the patient received HT within 6 months of surgery. Adjuvant RT was defined if patients received 40‐65Gy of EBRT to the breast within 6 months of surgery.^15^ Patients were classified as receiving either no adjuvant treatment, HT alone, RT alone, or HT and RT. The primary outcome of interest was overall survival after surgery. The NCDB recorded the number of months of follow‐up after diagnosis and the patient’s vital status at that time. Survival from surgery was calculated using the survival from diagnosis and the time from diagnosis to surgery.

### 2.4 Statistical analysis

All variables were compared across adjuvant treatment type using χ^2^ tests. A multivariable multinomial logistic regression was used to estimate Odds Ratios (OR) and 95% Confidence Intervals (CI) to assess covariates associated with adjuvant treatment type. Kaplan‐Meier curves and log‐rank tests were used to examine and compare univariate overall 5‐/10‐year survival across adjuvant treatment types. Hazards Ratios (HR) and 95% CIs were obtained from multivariable Cox proportional hazards models to assess the association between treatment and overall survival (OS), adjusted for confounding. This analysis was also stratified by age group. Change in use of HT and RT was assessed over time descriptively, as a percentage of the sample for each year of study.

Multivariable analyses were conducted only among the subset of patients who received any adjuvant treatment and were adjusted for age at diagnosis, race, ethnicity, primary insurance, median income for the patient’s ZIP code of residence, Charlson‐Deyo comorbidity score, patient’s distance from the reporting facility, reporting facility region and type, year of diagnosis, laterality, stage, tumor grade, histology, and number of regional lymph nodes examined. Those with missing values were excluded from these analyses.

A propensity‐matched analysis was conducted among the subset of patients who received RT alone or HT alone, in order to directly compare OS among these patients. A propensity score predicting receipt of HT alone (compared to RT alone) was calculated based on all covariates, and patients were matched 1:1 using the greedy algorithm, within each age group, in order to achieve balance.^16^ A Cox proportional hazards model was used to compare OS, accounting for matching.

## 3 RESULTS

We identified 130,194 patients who met our selection criteria; 12.8% received no adjuvant treatment, 18.1% received HT alone; 14.0% received RT alone, and 55.1% received both RT and HT. Those who received both RT and HT were significantly younger than those treated with HT or RT alone and those who received no adjuvant treatment (10.4% ≥80 years, compared to 23.3%, 33.6%, and 42.4%, respectively). Patients treated with HT alone were less likely to have private insurance (10.8%, compared to 15.0% for RT alone and 15.5% for RT and HT), more likely to have comorbidities (5.0% with score ≥2, compared to 3.2% for RT alone and RT and HT), to live further from the reporting facility (6.7% >50 miles, compared to 4.2% for RT alone and 4.9% for RT and HT) and be treated at an academic facility (31.3%, compared to 25.6% for RT alone, and 26.9% for RT and HT). See Table 1 for a complete comparison of adjuvant treatment types.

**Table 1:** Baseline characteristics according to adjuvant treatment type

	No Adjuvant Treatment^a^	Hormone Therapy Alone	Radiotherapy Alone	Hormone Therapy and Radiotherapy	
*Variable*	(n=16,689) N (%)	(n=23,548) N (%)	(n=18,220) N (%)	(n=71,737) N (%)	P‐value^b^
Age (years)					<.0001
65‐69	2473 (14.8)	3019 (12.8)	5149 (28.3)	29582 (41.2)	
70‐74	3411 (20.4)	6147 (26.1)	4577 (25.1)	21101 (29.4)	
75‐79	3733 (22.4)	6461 (27.4)	4253 (23.3)	13608 (19.0)	
≥80	7072 (42.4)	7921 (33.6)	4241 (23.3)	7446 (10.4)	
Race					0.0001
White	15179 (91.0)	21353 (90.7)	16557 (90.9)	65198 (90.9)	
Black	982 (5.9)	1559 (6.6)	1096 (6.0)	4293 (6.0)	
Other	365 (2.2)	495 (2.1)	434(2.4)	1791 (2.5)	
Missing	163 (1.0)	141 (0.6)	133 (0.7)	455 (0.6)	
Hispanic					<.0001
No	15207 (91.1)	22027 (93.5)	16678 (91.5)	66503 (92.7)	
Yes	639 (3.8)	530 (2.3)	434 (2.4)	1887 (2.6)	
Missing	843 (5.1)	991 (4.2)	1108 (6.1)	3347 (4.7)	
Insurance					<.0001
Uninsured	67 (0.4)	66 (0.3)	48 (0.3)	220 (0.3)	
Private Insurance	1926 (11.5)	2544 (10.8)	2727 (15.0)	11151 (15.5)	
Medicare/Medicaid	14416 (86.4)	20598 (87.5)	15142 (83.1)	59444 (82.9)	
Other Government	60 (0.4)	90 (0.4)	73 (0.4)	253 (0.4)	
Missing	220 (1.3)	250 (1.1)	230 (1.3)	669 (0.9)	
Median Income ($)^c^					<.0001
<48,000	5973 (35.8)	8783 (37.3)	6162 (33.8)	24678 (34.4)	
≥48,000	10537 (63.1)	14640 (62.2)	11917 (65.4)	46724 (65.1)	
Missing	179 (1.1)	125 (0.5)	141 (0.8)	335 (0.5)	
Charlson‐Deyo Comorbidity Score					<.0001
0‐1	15862 (95.0)	22375 (95.0)	17630 (96.8)	69409 (96.8)	
≥2	827 (5.0)	1173 (5.0)	590 (3.2)	2328 (3.2)	
Distance from Reporting Facility (Miles)					<.0001
≤50	15503 (92.9)	21870 (92.9)	17318 (95.0)	67891 (94.6)	
>50	1009 (6.0)	1568 (6.7)	768 (4.2)	3515 (4.9)	
Missing	177 (1.1)	110 (0.5)	134 (0.7)	331 (0.5)	
Facility Location					<.0001
Northeast	3594 (21.5)	5397 (22.9)	4102 (22.5)	18074 (25.2)	
South	6375 (38.2)	8330 (35.4)	5397 (29.6)	21248 (29.6)	
Central	3536 (21.2)	6453 (27.4)	4705 (25.8)	21752 (30.3)	
West	3184 (19.1)	3368 (14.3)	4016 (22.0)	10663 (14.9)	
Facility Type					<.0001
Non‐Academic	12156 (72.8)	16171 (68.7)	13548 (74.4)	52443 (73.1)	
Academic	4533 (27.2)	7377 (31.3)	4672 (25.6)	19294 (26.9)	
Year of Diagnosis					<.0001
2004‐2008	5697 (34.1)	4277 (18.2)	6771 (37.2)	16306 (22.7)	
2009‐2013	7414 (44.4)	11060 (47.0)	8352 (45.8)	35229 (49.1)	
2014‐2015	3578 (21.4)	8211 (34.9)	3097 (17.0)	20202 (28.2)	
Laterality					0.0013
Left	8435 (50.5)	12137 (51.5)	9095 (49.9)	35993 (50.2)	
Right	≥10^d^	≥10^d^	≥10^d^	35732 (49.8)	
Missing	<10^d^	<10^d^	<10^d^	12 (0.02)	
Tstage					<.0001
Tmi	277 (1.7)	242 (1.0)	379 (2.1)	817 (1.1)	
T1a/b	8907 (53.4)	11607 (49.3)	10347 (56.8)	32857 (45.8)	
T1c	6354 (38.1)	10702 (45.4)	6309 (34.6)	34831 (48.6)	
T1 Not Otherwise Specified	1151 (6.9)	997 (4.2)	1185 (6.5)	3232 (4.5)	
Grade					<.0001
Well Differentiated	7198 (43.1)	9927 (42.2)	7888 (43.3)	27322 (38.1)	
Moderately differentiated	7228 (43.3)	10794 (45.8)	7741 (42.5)	34479 (48.1)	
Poorly differentiated/anaplastic	1360 (8.1)	1691 (7.2)	1472 (8.1)	6324 (8.8)	
Missing	903 (5.4)	1136 (4.8)	1119 (6.1)	3612 (5.0)	
Histology					<.0001
Ductal	12009 (72.0)	17366 (73.7)	13445 (73.8)	52900 (73.7)	
Lobular	3003 (18.0)	4433 (18.8)	3189 (17.5)	14444(20.1)	
Other	1677 (10.0)	1749 (7.4)	1586 (8.7)	4393 (6.1)	
Regional Lymph Nodes Examined					<.0001
None	2463 (14.8)	2238 (9.5)	730 (4.0)	1432 (2.0)	
1‐5	12670 (75.9)	19316 (82.0)	15429 (84.7)	62676 (87.4)	
>5	1446 (8.7)	1854 (7.9)	1907 (10.5)	7124 (9.9)	
Missing	110 (0.7)	140 (0.6)	154 (0.8)	505 (0.7)	

^a^ Excluded from subsequent multivariable analyses.

^b^ p‐value from χ^2^ test, based on non‐missing values.

^c^ ZIP code level data, based on patient’s residence.

^d^ exact cell sizes masked to protect against identification of patients.

**Figure 2:**
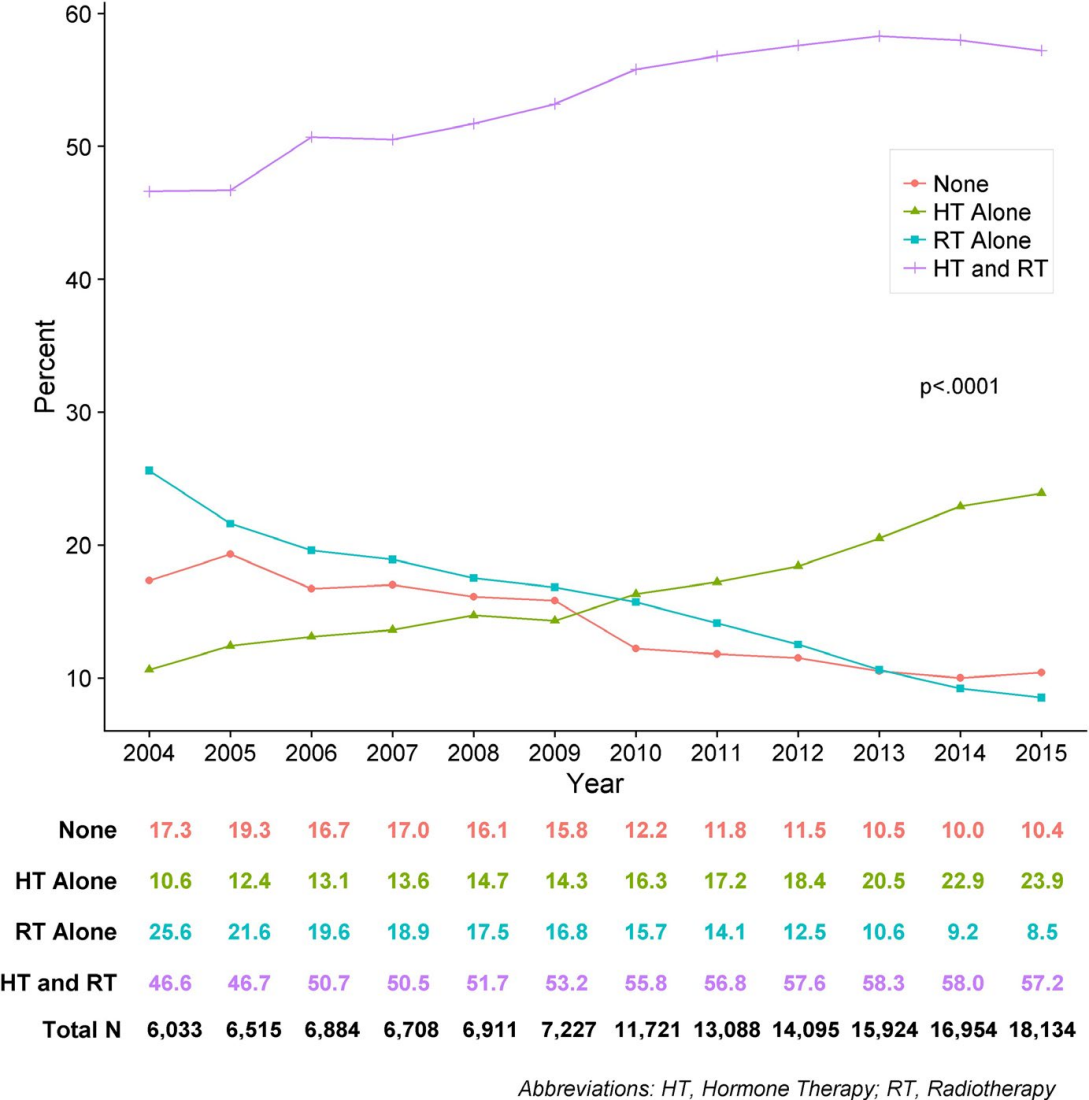
Trends in adjuvant treatment type over time

Over time, the proportion of patients receiving any adjuvant treatment increased from 82.7% in 2004 to 89.6% in 2015. There was an increase in the utilization of HT alone and HT and RT, while the use of RT alone declined (p<0.0001) (Figure 2).

After adjusting for all covariates among patients who received any adjuvant therapy (Table 2), patients treated with RT alone or RT and HT were more likely to be younger, with private insurance, higher income, and have lower comorbidity score than patients treated with HT alone. They were also more often diagnosed in earlier years of the study period and received treatment at a non‐academic facility that was ≤50 miles away from their residence. Patients with larger primary tumors (pT1c) more often received RT and HT and less often received RT alone, and those with poorly differentiated tumors more often received both combination RT and HT and RT alone.

**Table 2:** Predictors of adjuvant therapy type (compared to hormonal therapy alone) among patients receiving treatment (n=99,927)

	Radiotherapy Alone	Hormone Therapy and Radiotherapy
*Covariates*	OR_adj_ ^b^ (95% CI)	OR_adj_ ^b^ (95% CI)
Age (years)		
65‐69	1.00 (ref)	1.00 (ref)
70‐74	0.43 (0.40‐0.46)	0.34 (0.32‐0.36)
75‐79	0.36 (0.33‐0.38)	0.20 (0.19‐0.21)
≥80	0.30 (0.28‐0.32)	0.09 (0.08‐0.09)
Race		
White	1.00 (ref)	1.00 (ref)
Black	1.08 (0.98‐1.18)	0.98 (0.91‐1.05)
Other	0.93 (0.81‐1.08)	1.10 (0.98‐1.24)
Hispanic		
Yes vs. No	1.02 (0.89‐1.18)	1.12 (1.00‐1.25)
Insurance		
Medicare/Medicaid	1.00 (ref)	1.00 (ref)
Other Government	1.22 (0.87‐1.71)	1.04 (0.80‐1.36)
Private Insurance	1.13 (1.06‐1.20)	1.10 (1.05‐1.16)
Uninsured	0.93 (0.62‐1.40)	0.94 (0.69‐1.28)
Median Income ($)^a^		
≥48,000 vs. <48,000	1.10 (1.05‐1.16)	1.07 (1.03‐1.11)
Charlson‐Deyo Comorbidity Score		
≥2 vs. 0‐1	0.71 (0.64‐0.79)	0.67 (0.62‐0.73)
Distance from reporting Facility (miles)		
>50 vs. ≤50	0.60 (0.54‐0.67)	0.66 (0.61‐0.71)
Facility Location		
Northeast	1.00 (ref)	1.00 (ref)
Central	0.97 (0.91‐1.03)	1.01 (0.96‐1.05)
South	0.83 (0.78‐0.88)	0.68 (0.65‐0.71)
West	1.56 (1.45‐1.67)	0.82 (0.78‐0.87)
Facility Type		
Academic vs. Non‐Academic	0.75 (0.71‐0.78)	0.69 (0.67‐0.72)
Year of Diagnosis		
2004‐2008	1.00 (ref)	1.00 (ref)
2009‐2013	0.44 (0.42‐0.46)	0.73 (0.69‐0.76)
2014‐2015	0.21 (0.19‐0.22)	0.49 (0.47‐0.52)
Laterality		
Right vs. Left	1.07 (1.02‐1.11)	1.05 (1.02‐1.09)
Stage		
Tmi	1.00 (ref)	1.00 (ref)
T1a/b	0.61 (0.50‐0.76)	0.97 (0.80‐1.17)
T1c	0.41 (0.33‐0.51)	1.21 (1.00‐1.46)
T1 Not Otherwise Specified	0.54 (0.43‐0.68)	1.02 (0.83‐1.26)
Grade		
Well Differentiated	1.00 (ref)	1.00 (ref)
Moderately Differentiated	0.98 (0.94‐1.03)	1.21 (1.17‐1.25)
Poorly Differentiated/Anaplastic	1.24 (1.14‐1.34)	1.53 (1.43‐1.63)
Histology		
Ductal	1.00 (ref)	1.00 (ref)
Lobular	1.00 (0.95‐1.06)	1.12 (1.07‐1.17)
Other	1.16 (1.07‐1.26)	0.93 (0.87‐0.99)
Regional Lymph Nodes Examined		
None	1.00 (ref)	1.00 (ref)
1‐5	2.53 (2.29‐2.79)	3.26 (3.01‐3.53)
>5	2.65 (2.36‐2.99)	3.52 (3.20‐3.88)

Abbreviations: CI, Confidence Interval; OR, Odds Ratio.

n=99,297.

reference group: Hormone Therapy Alone.

^a^ ZIP code level data, based on patient’s residence.

^b^ Adjusted for all variables listed.

**Figure 3:**
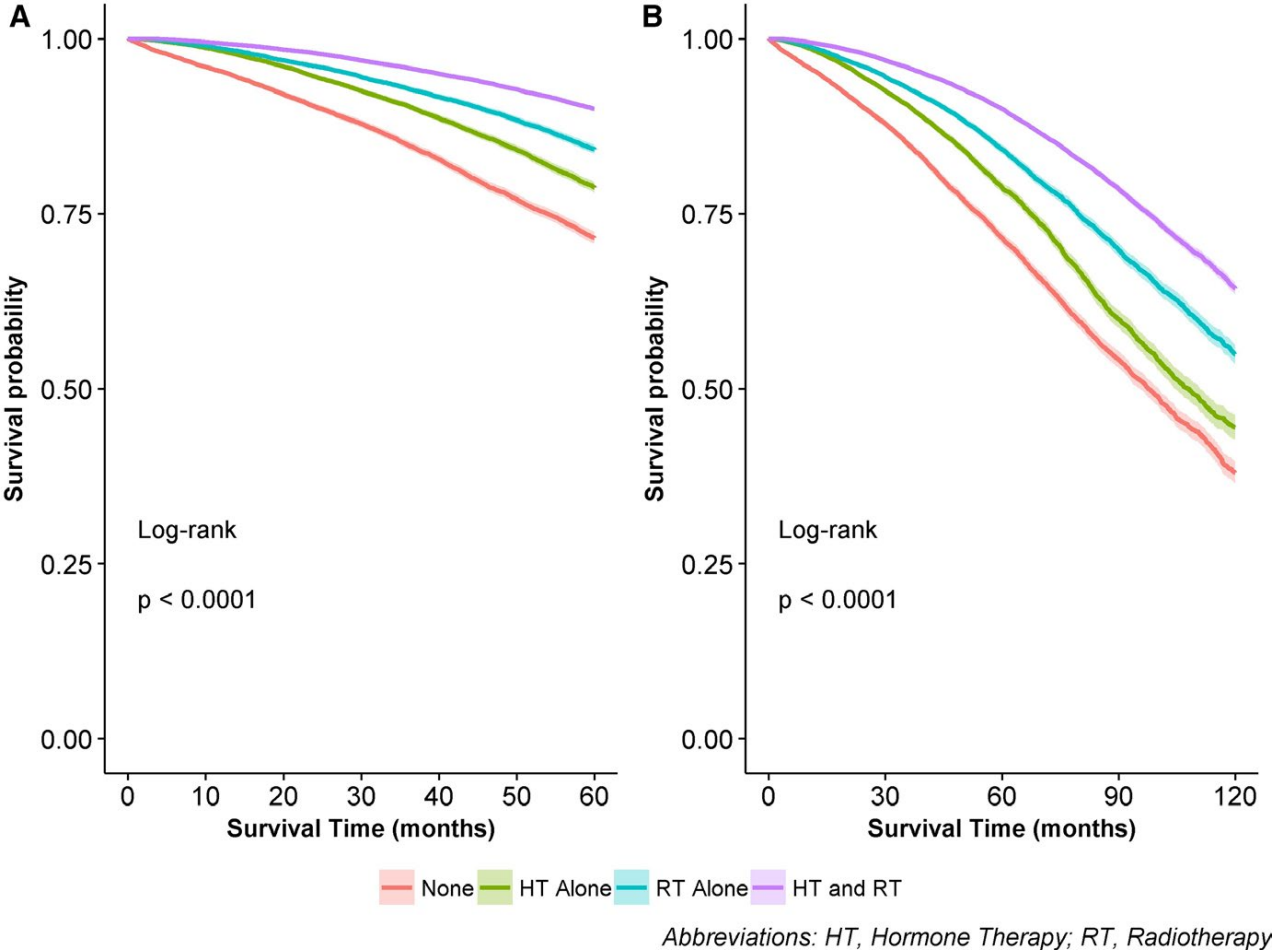
Overall survival at 5 years (A) and 10 years (B) according to adjuvant treatment type on univariate analysis

The univariate 5‐/10‐year OS was 71.6%/38.0% for those with no adjuvant treatment; 78.7%/44.5% for those with HT alone; 84.2%/54.9% for those with RT alone; and 90.0%/64.3% for those with RT and HT (p<0.0001 for both) (Table 3, Figure 3). After adjusting for all covariates among patients who received any adjuvant therapy, 5‐ and 10‐year OS was significantly better for those with RT alone (HR_adj_: 0.84, 95% CI: 0.79‐0.90; HR_adj_: 0.86, 95% CI: 0.82‐0.91, respectively) and RT and HT (HR_adj_: 0.61, 95% CI: 0.57‐0.64; HR_adj_: 0.67, 95% CI: 0.64‐0.70, respectively), compared to those with HT alone (Table 3). Other variables associated with improved survival were younger age, Hispanic ethnicity, private insurance, higher income, lower comorbidity score, greater distance from the reporting facility, diagnosis in an academic facility, earlier stage, well differentiated tumors, and more lymph nodes examined (data not shown). Results were similar when stratified by age. In every age group, 5‐/10‐ year OS was better for those patients who received either RT alone or RT and HT, compared to those who received HT alone, after adjusting for confounding (data not shown).

**Table 3:** Univariate and multivariable 5‐/10‐year overall survival by adjuvant treatment

	5 Year Survival		
	Survival Percent (95% CI)	*P*	Adj HR^b^ (95% CI)
No Adjuvant Treatment^a^	71.6 (70.7‐72.4)		X
Hormone Therapy Alone	78.7 (77.9‐79.5)		1.00 (ref)
Radiotherapy Alone	84.2 (83.6‐84.8)		0.84 (0.79‐0.90)
Hormone Therapy and Radiotherapy	90.0 (89.7‐90.3)	<.0001	0.61 (0.57‐0.64)

	10 Year Survival		
	Survival Percent (95% CI)	*P*	Adj HR^b^ (95% CI)
No Adjuvant Treatment^a^	38.0 (36.4‐39.6)		X
Hormone Therapy Alone	44.5 (42.7‐46.3)		1.00 (ref)
Radiotherapy Alone	54.9 (53.5‐56.3)		0.86 (0.82‐0.91)
Hormone Therapy and Radiotherapy	64.3 (63.5‐65.2)	<.0001	0.67 (0.64‐0.70)

Abbreviations: Adj, Adjusted; CI, Confidence Interval; HR, Hazard Ratio.

^a^ Group with no adjuvant treatment excluded from the multivariable analysis.

^b^ Adjusted for age at diagnosis, race, ethnicity, insurance, income, Charlson‐Deyo comorbidity score, distance from the reporting facility, facility location region and type, year of diagnosis, laterality, stage, grade, histology, and number of regional lymph nodes examined (n=84,717).

The propensity matched cohort of patients who received RT alone or HT alone (n=21,326) was well balanced on all covariates (p‐value from McNemar’s test ranging from 0.1638 to 1, eTable 1). Those receiving RT alone had significantly better 5‐/10‐year survival, compared to those receiving HT alone (HR_adj_: 0.84, 95% CI: 0.78‐0.92; HR_adj_: 0.87, 95% CI: 0.81‐0.94, respectively) (Table 4).

**Table 4:** Overall survival by adjuvant treatment time in a propensity‐matched cohort (n=21,326)*

	5 Year Survival		
	Survival Percent (95% CI)	*P*	HR (95% CI)
Hormone Therapy Alone	80.2 (79.3‐81.2)		1.00 (ref)
Radiotherapy Alone	83.0 (82.2‐83.9)	<.0001	0.84 (0.78‐0.92)

	10 Year Survival		
	Survival Percent (95% CI)	*P*	HR (95% CI)
Hormone Therapy Alone	46.7 (44.5‐48.8)		1.00 (ref)
Radiotherapy Alone	49.3 (47.2‐51.4)	0.0002	0.87 (0.81‐0.94)

Abbreviations: CI, Confidence Interval; HR, Hazard Ratio.

^*^Propensity‐matched on age at diagnosis, race, ethnicity, insurance, income, Charlson‐Deyo comorbidity score, distance from the reporting facility, facility location region and type, year of diagnosis, laterality, stage, grade, histology, and number of regional lymph nodes examined.

## 4 DISCUSSION

This is a large, cancer registry‐based analysis of older patients with early‐stage ER positive breast cancer that assesses trends in adjuvant management and compares survival outcomes based on adjuvant therapy. Multiple patient, facility, and tumor variables were identified that were significantly associated with adjuvant management. Survival analyses demonstrated that compared to HT alone, patients who received combination HT and RT had the best unadjusted and adjusted survival outcomes. Comparison of single modality therapy showed improved unadjusted and adjusted survival among patients who received RT alone. This finding was confirmed in a well‐balanced propensity matched cohort of patients who received single modality therapy. This study represents the largest known analysis to date that examines the role of RT relative to HT in this patient population.

To date, the randomized trials that have looked at use of monotherapy in the adjuvant setting focused on eliminating RT from the treatment paradigm. CALGB9343 looked at women 70 years or older with clinical stage I (98% T1N0M0), ER positive breast cancers who underwent lumpectomy. Patients were randomized to Tamoxifen with RT or Tamoxifen only. This trial showed a significant decrease in the rate of local failures at 10 years with the addition of RT (10% vs. 2%) but no significant difference in time to distant metastases, breast cancer‐specific survival, or overall survival. ^3^, ^4^. The PRIME II study included women age 65 or older with T1‐T2, N0, ER positive disease. This trial similarly compared Tamoxifen with RT to Tamoxifen alone after BCS and showed a significant decrease in 5‐year in‐breast tumor recurrence with RT (4.1% vs. 1.3%). There was no difference in regional recurrence, distant metastases, or overall survival.^5^ Despite the consistent local control benefit with RT, these results are often interpreted to support the omission of RT.

While hormonal therapy can improve local, distant, and contralateral disease control, compliance with long‐term therapy due to toxicity remains a well‐documented issue.^8^, ^9^, ^10^ Perhaps patients with small, hormone receptor positive tumors, which are generally thought of as biologically favorable, do not have a significant distant relapse risk that necessitates systemic treatment. In fact, women over the age of 70 are more likely to have lower Oncotype DX recurrence score, which is associated with less distant metastasis risk.^17^ With regards to contralateral breast cancer, it has been demonstrated that the most important prognostic factor is age at first diagnosis,^18^ again suggesting that this benefit of endocrine therapy is more important in the younger patient population. By comparison, modern radiotherapy regimens allow for the completion of whole breast irradiation in only 3‐4 weeks with low rates of toxicity.^11^, ^19^, ^20^, ^21^

This study has several important limitations. It is a retrospective analysis using data from participating institutions, which mainly represent hospital‐based practices accredited by the American College of Surgeons Commission on Cancer. Errors in reporting or coding cannot be accounted for. The only long‐term endpoint available in the National Cancer Data Base is overall survival. The NCDB does not contain data on cosmesis or patient‐reported quality of life outcomes. While our cohort includes patients diagnosed between 2004 and 2015, the NCDB did not reliably account for HER2 status until 2010. We have attempted to address this by excluding all patients who received chemotherapy and immunotherapy, it is possible that a small minority of patients were HER2 positive. Additionally, the nature of the available data does not allow us to capture the length of time that patients received adjuvant endocrine therapy or data on compliance. This is a particularly important limitation when comparing outcomes based on adjuvant therapy received. It is likely, however, that these data are more representative of outcomes based off of true adherence to therapy in the community setting than that seen in closely monitored randomized controlled trials. Finally, despite our best efforts to account for potential confounders with multivariable analyses and propensity score matching, it is impossible to completely eliminate the risk of selection bias. Specifically, in a cohort of women age 65+ with low‐risk breast cancer, the impact of underlying co‐morbidities on survival outcomes can be quite significant. Unfortunately, in the NCDB, the only measure of co‐morbidities is the Charlson‐Deyo score, which likely does not capture the true impact of underlying conditions on survival in this particular patient population. It is possible that our findings may, in part, be a result of providers correctly identifying patients with fewer co‐morbidities and longer life expectancy and therefore recommending more aggressive therapy (HT and RT). In the absence of randomized data, however, the present analysis is the largest known study to compare outcomes based on adjuvant therapy in this patient population.

## 5 CONCLUSIONS

Older women with early stage ER+ breast cancer who received adjuvant HT and RT had the best survival compared to HT alone or RT alone. However, multivariable assessment of single‐modality therapy showed that RT alone had higher rates of OS at 5 and 10 years compared to HT alone. This was confirmed in a well‐balanced propensity matched cohort. The findings from this large cancer registry‐based analysis support the prospective comparison of single‐modality therapies in a randomized setting.

## CONFLICTS OF INTEREST

No potential conflict of interest was reported by any of the authors

## AUTHOR CONTRIBUTIONS

Sachin R Jhawar: Conceptualization, formal analysis, interpretation of data, writing, review, and editing. Naomi Alpert: Data curation, methodology, writing, review, and editing. Emanuela Taioli: Data curation, methodology, writing, review, and editing. Mutlay Sayan: Analysis, writing, review, and editing. Jose Bazan: Analysis, writing, review, and editing. Ko Un Park: Analysis, writing, review, and editing. Daniel Stover: Analysis, writing, review, and editing. Mathew Cherian: Analysis, writing, review, and editing. Julia White: Analysis, writing, review, and editing. Bruce Haffty: Analysis, writing, review, and editing. Nish Ohri: Conceptualization, formal analysis, interpretation of data, writing, review, and editing.

## Supporting information

Table S1

## Data Availability

The data that support the findings of this study are available from the National Cancer Database (NCDB). Restrictions apply to the availability of these data, which can be requested with the permission of the NCDB.
