# Supplementary material for: Adjuvant radiation therapy alone is associated with improved overall survival compared to hormonal therapy alone in older women with estrogen receptor positive early stage breast cancer
Source: Cancer Med. 2020 Sep 17;9(22):8345–54. doi: 10.1002/cam4.3443 (PMC7666745; doi:10.1002/cam4.3443)
Supplement: Supplementary file 1 — Table S1 [file CAM4-9-8345-s001.docx]

**eTable 1. Baseline characteristics of the propensity-matched cohort according to adjuvant treatment type (n=21,326).**

| ***Variable*** | **Hormone Therapy Alone**  **(n=10,663)**  **N (%)** | **Radiotherapy Alone**  **(n=10,663)**  **N (%)** | **P-value^** |
| --- | --- | --- | --- |
| Age (years) |  |  | 1.0 |
| 65-69 | 2033 (19.1) | 2033 (19.1) |  |
| 70-74 | 2769 (26.0) | 2769 (26.0) |  |
| 75-79 | 2751 (25.8) | 2751 (25.8) |  |
| ≥80 | 3110 (29.2) | 3110 (29.2) |  |
| Race |  |  | 0.7589 |
| White | 9851 (92.4) | 9870 (92.6) |  |
| Black | 612 (5.7) | 592 (5.6) |  |
| Other | 200 (1.9) | 201 (1.9) |  |
| Hispanic |  |  | 0.8782 |
| No | 10433 (97.8) | 10430 (97.8) |  |
| Yes | 230 (2.2) | 233 (2.2) |  |
| Insurance |  |  | 0.7410 |
| Uninsured | 32 (0.3) | 31 (0.3) |  |
| Private Insurance | 1314 (12.3) | 1354 (12.7) |  |
| Medicare/Medicaid | 9284 (87.1) | 9240 (86.7) |  |
| Other Government | 33 (0.3) | 38 (0.4) |  |
| Median Income ($)^ǂ^ |  |  | 0.3184 |
| <48,000 | 3762 (35.3) | 3809 (35.7) |  |
| ≥48,000 | 6901 (64.7) | 6854 (64.3) |  |
| Charlson-Deyo Comorbidity Score |  |  | 0.7741 |
| 0-1 | 10276 (96.4) | 10269 (96.3) |  |
| ≥2 | 387 (64.7) | 394 (3.7) |  |
| Distance from Reporting Facility (Miles) |  |  | 0.5801 |
| ≤50 | 10163 (95.3) | 10148 (95.2) |  |
| >50 | 500 (4.7) | 515 (4.8) |  |
| Facility Location |  |  | 0.1638 |
| Northeast | 2440 (22.9) | 2563 (23.1) |  |
| South | 3579 (33.6) | 3574 (33.5) |  |
| Central | 1694 (15.9) | 2726 (25.6) |  |
| West | 1950 (18.3) | 1900 (17.8) |  |
| Facility Type |  |  | 0.4675 |
| Non-Academic | 7805 (73.2) | 7837 (73.5) |  |
| Academic | 2858 (26.8) | 2826 (26.5) |  |
| Year of Diagnosis |  |  | 0.4867 |
| 2004-2008 | 3112 (29.2) | 3115 (29.2) |  |
| 2009-2013 | 6177 (57.9) | 6217 (58.3) |  |
| 2014-2015 | 1374 (12.9) | 1900 (17.8) |  |
| Laterality |  |  | 0.5893 |
| Left | 5350 (50.2) | 5376 (50.4) |  |
| Right | 5313 (49.8) | 5287 (49.6) |  |
| Tstage |  |  | 0.9137 |
| Tmi | 104 (1.0) | 105 (1.0) |  |
| T1a/b | 5697 (53.4) | 5751 (53.9) |  |
| T1c | 4256 (39.9) | 4198 (39.4) |  |
| T1 Not Otherwise Specified | 606 (5.7) | 609 (5.7) |  |
| Grade |  |  | 0.7589 |
| Well Differentiated | 4805 (45.1) | 4831 (45.3) |  |
| Moderately differentiated | 4986 (46.8) | 4954 (46.5) |  |
| Poorly differentiated/anaplastic | 872 (8.2) | 878 (8.2) |  |
| Histology |  |  | 0.2782 |
| Ductal | 8047 (75.5) | 8058 (75.6) |  |
| Lobular | 1868 (17.5) | 1813 (17.0) |  |
| Other | 748 (7.0) | 792 (7.4) |  |
| Regional Lymph Nodes Examined |  |  | 0.6696 |
| None | 529 (5.0) | 551 (5.2) |  |
| 1-5 | 9150 (85.8) | 9107 (85.4) |  |
| >5 | 984 (9.2) | 1005 (9.4) |  |
| *^ p-value from McNemar’s test*  *^ǂ^ZIP code level data, based on patient’s residence* | | | |
